# Vagal Nerve Stimulation Rapidly Activates Brain-Derived Neurotrophic Factor Receptor TrkB in Rat Brain

**DOI:** 10.1371/journal.pone.0034844

**Published:** 2012-05-01

**Authors:** Havan Furmaga, Flavia Regina Carreno, Alan Frazer

**Affiliations:** 1 Department of Pharmacology, University of Texas Health Science Center, San Antonio, Texas, United States of America; 2 South Texas Veterans Health Care System, Audie L. Murphy Division, San Antonio, Texas, United States of America; INSERM, UMR-S747, France

## Abstract

**Background:**

Vagal nerve stimulation (VNS) has been approved for treatment-resistant depression. Many antidepressants increase expression of brain-derived neurotrophic factor (BDNF) in brain or activate, via phosphorylation, its receptor, TrkB. There have been no studies yet of whether VNS would also cause phosphorylation of TrkB.

**Methods:**

Western blot analysis was used to evaluate the phosphorylation status of TrkB in the hippocampus of rats administered VNS either acutely or chronically. Acute effects of VNS were compared with those caused by fluoxetine or desipramine (DMI) whereas its chronic effects were compared with those of sertraline or DMI.

**Results:**

All treatments, given either acutely or chronically, significantly elevated phosphorylation of tyrosines 705 and 816 on TrkB in the hippocampus. However, only VNS increased the phosphorylation of tyrosine 515, with both acute and chronic administration causing this effect. Pretreatment with K252a, a nonspecific tyrosine kinase inhibitor, blocked the phosphorylation caused by acute VNS at all three tyrosines. Downstream effectors of Y515, namely Akt and ERK, were also phosphorylated after acute treatment with VNS, whereas DMI did not cause this effect.

**Conclusion:**

VNS rapidly activates TrkB phosphorylation and this effect persists over time. VNS-induced phosphorylation of tyrosine 515 is distinct from the effect of standard antidepressant drugs.

## Introduction

Neurotrophins, particularly brain-derived neurotrophic factor (BDNF), are key molecules regulating neuronal survival, development, function and plasticity [1]. BDNF binding to its receptor, TrkB [2], results in receptor dimerization and autophosphorylation in the kinase domain of its cytosolic region, followed by activation of various signaling pathways. Specific signaling is promoted by phosphorylation of other tyrosine residues so as to create docking sites for adapter proteins that couple the receptors to intracellular signal transduction mechanisms. Autophosphorylation of the TrkB catalytic domain at tyrosine 705 is considered a critical step in TrkB receptor activation, which further regulates the phosphorylation and activation of other tyrosines, of which tyrosines 515 and 816 are most extensively studied [2], [3]. Phosphorylation of Y515 leads to activation of Ras/mitogen activated protein kinase (Ras/MAPK) and phosphoinositide 3-kinases (PI3K) signaling pathways, whereas phosphorylation of tyrosine 816 induces activation of the phospholipase C- γ1 (PLC-γ1)-mediated signaling pathway [2].

The neurotrophic hypothesis of depression and antidepressant action arose from evidence suggesting that BDNF levels are reduced in mood disorders [4], [5], [6] and that chronic antidepressant treatment enhances BDNF expression and signaling [7], [8], [9]. Although chronic treatment with antidepressants is required to increase the expression of BDNF message [10], [11] or protein [6], [12], acute antidepressant treatment can rapidly activate TrkB receptors. Available evidence indicate that shortly after single administration of different classes of antidepressants to mice, there was increased phosphorylation of TrkB receptors in cerebral cortex and the hippocampus [13], [14]. Two tyrosines were found to be phosphorylated, Y705 and Y816. Moreover, Saarelainen et al. [14] found that signaling *via* the TrkB receptor is required for a behavioral response typically induced by antidepressants. In particular, they showed that administration of antidepressants to transgenic mice over-expressing the truncated TrkB-T1 isoform, which lacks the functional cytosolic signaling domain, does not produce less immobility in the forced swim test (FST).

Vagal nerve stimulation (VNS) has been approved by the Food and Drugs Administration (FDA) for treatment-resistant epilepsy (1997) and treatment-resistant depression (TRD) (2005). Over long time periods (e.g., one year) it has been shown to be effective in reducing the severity of symptoms in patients with TRD [15], [16], [17], [18]. When given repeatedly to animals, VNS has been shown to activate both noradrenergic and serotonergic cell body areas [19], [20]. Behaviorally, Krahl et al. [21] reported that 30 min per day of VNS for 4 days significantly reduced immobility in the FST, i.e., it had antidepressant-like activity. We have shown recently that its administration chronically (14 days) also causes antidepressant-like activity in the FST that is not seen in rats with lesions of serotonergic neurons [22]. Given the possible involvement of BDNF in antidepressant action, some investigators have begun to examine the effect of VNS on BDNF message. VNS given for just three hours increased message for BDNF in rat hippocampus and cerebral cortex [23] and this has also been seen in hippocampus after chronic VNS [24]. There have been no studies though examining the effect of either acute or repeated administration of VNS on the phosphorylation of TrkB in rat brain. We hypothesized that both acute and repeated VNS would increase the phosphorylation of the tyrosines at Y705 and Y816, similar to what has been found with antidepressants. This was observed but in addition another tyrosine, Y515, was found to be phosphorylated as well.

## Materials and Methods

Experiments were carried out using adult male Sprague-Dawley rats, 250–350 g (Harlan). Rats were group housed and maintained in a temperature-controlled environment on a 14∶10 h light-dark cycle and had access to food and water *ad libitum*. Experimental protocols were approved by the IACUC in accordance with the guidelines of the Public Health Service, American Physiological Society, and the Society for Neuroscience.

### Implantation of vagal nerve stimulators

Vagus nerve electrodes were implanted on the vagus nerve under aseptic conditions. The surgical procedure was similar to that described by Cunningham et al. [25] except that the anesthetic was a mixture of 75 mg/kg ketamine and 0.5 mg/kg medetomidine. Briefly, the coil electrode was placed around the left cervical vagus nerve and carotid sinus ventral to the carotid bifurcation. The bipolar stimulating electrode was configured with the cathode at the proximal lead and the anode at the distal lead to preferentially direct action potential propagation toward the central nervous system by creating an anodal block at the distal lead. The electrodes were connected to a stimulator pack (Cyberonics, Inc., Houston TX) and placed in a subcutaneous pouch on the back of the rat. Rats that received VNS were instrumented with an operational stimulator pack that was programmed by a handheld computer. Control rats received a dummy simulator pack that was the same size and weight (48 mm ×33 mm ×7.1 mm; 16 g). The rat was injected with penicillin immediately post-op and topical antibiotic applied to the wound. Rats were monitored during recovery under a heat lamp until fully ambulatory. Beginning 7 days after surgery, VNS was turned on for either 2 hours (acute) or 14 days (chronic). The stimulation paradigm consisted of one burst of 20 Hz, 250 µsec pulse width, 250 µA current for 30 sec every 5 min. These parameters are the initial parameters used in clinical studies [15],[16]. Also, we found that doubling the current used to 500 µA caused autonomic changes in blood pressure and heart rate (data not shown). For both the acute and the chronic studies, VNS stimulation occurred until the rats were sacrificed.

### Implantation of Osmotic Minipumps

This was done exactly as described previously [26] except that the pumps delivered fluid intraperitoneally rather than subcutaneously. One day prior to surgery, osmotic minipumps delivering 5 µl/h (Model 2ML2, DURECT Corporation, Cupertino, CA) were filled with drug or vehicle, filtered through 0.9-µm nitrocellulose filters (Millipore, Bedford, MA) using a sterile technique in an air-filtered hood. Drug solution concentrations were determined based on the mean rat weight over the 14 days of treatment. Either a dose of 7.5 mg/kg/day of sertraline or 10 mg/kg/day desipramine produced serum concentrations in the therapeutic range [26], [27]. The vehicle solution consisted of 10% EtOH/0.9% NaCl [28]. Minipumps were stored in sealed containers filled with sterile saline in a 30–32°C incubator to prime the pumps until the time of surgery. At the time of surgery, rats were anesthetized with an intramuscular injection of a mixture of 75 mg/kg ketamine and 0.5 mg/kg medetomidine. A drug-filled minipump was implanted intraperitoneally via a midline incision made on the lower abdomen. After the pump was implanted, the muscle and skin were sutured. The rat was injected with penicillin immediately post-op and topical antibiotic applied to the wound. Rats were monitored during recovery under a heat lamp until fully ambulatory.

### Intracerebroventricular (i.c.v) administration of K252a

Rats were administered K252a (Calbiochem, San Diego, CA). K252a is a nonspecific tyrosine kinase inhibitor [29] that has been shown to block autophosphorylation of TrkB and inhibit many biological functions of BDNF [30]. The total dose administered of either neurotoxin was 10 µg in 10 µl of saline or PBS containing 1% DMSO. When administered intracerebroventricularlyat this dose, Benmansour et al. [31] found that it blocked the effect of exogenously administered BDNF on serotonin clearance. Control rats were injected with vehicle. Rats were anesthetized with a mixture of 75 mg/kg ketamine and 0.5 mg/kg medetomidine and placed into a stereotaxic frame. K252a was administered i.c.vwith stereotaxic coordinates, AP, −0.8 mm from Bregma; ML, +1.4 mm from midline; DV, −4.0 mm from dura [31] using a 28-gauge stainless steel injector attached to a Bee Syringe Pump (Bioanalytical System Inc, West Lafayette, IN, USA) at a rate of 1 µL/min. Once the infusion was completed, the injector was left in place for an additional 5 min to allow diffusion of vehicle or K252a.

### Western blot analysis

Rats were sacrificed by rapid decapitation, brains quickly removed and hippocampi were dissected out on an ice-cooled dish. Samples were stored at −80°C until processed for Western blot analysis. Samples were homogenized in a lysis buffer (50 mM Tris, 1 mM EDTA, 0.35% NA Deoxycholate, 150 mM NaCl, 1% Igepal, H_2_O, and 10 µl of protease inhibitor mixture (Sigma) per 100 mg tissue), incubated on ice for 30 min and then centrifuged (13000 g, 15 min). Protein levels of the collected supernates were measured using the Bradford assay (Biorad, Hercules, CA). Proteins were separated in a SDS-PAGE gel and blotted onto a nitrocellulose membrane. Membranes were incubated at 4°C overnight with the following primary antibodies: anti-phoshoY515 (1∶1000 in 1% BSA in TBST, Abcam, Cambridge, MA), anti-phoshoY705 (1∶1000 in 1% BSA in TBST, Abcam, Cambridge, MA), anti-phoshoY816 (1∶4000 in 1% BSA in TBST, Abcam), and anti-TrkB (1∶10000 in 1% BSA in TBST, Neuromics, Edina, MN), anti-phoshoAkt (Ser473; 1∶1000 in 5% BSA in TBST, Cell Signaling Technology, Inc., Danvers, MA), anti-phoshoAkt (Thr308; 1∶1000 in 5% BSA in TBST, Cell Signaling Technology, Inc), anti-Akt (1∶500 in 1% BSA in TBST, Invitrogen Corporation, Carmarillo, CA), anti-p44/42 MAPK (Erk1/2; 1∶2000 in 5% BSA in TBST, Cell Signaling Technology, Inc.), anti-phosho-p42/44 MAPK (pERK1/2, Thr202/Tyr204; 1∶1000 in 5% BSA in TBST, Cell Signaling Technology, Inc.). Equal loading was confirmed using anti-β-actin (1∶100,000 in 1% BSA in TBST, Sigma), which was routinely used for normalization. Membranes were washed with TBS/0.1% Tween (TBST) and incubated with horseradish peroxidase conjugated secondary antibody (1∶5000 in 1% BSA in TBST, Sigma). Secondary antibodies were visualized using enhanced chemiluminescence kits (Pierce, Rockford, IL) followed by an exposure to X-ray film for detection.

Results were calculated as the ratio of phosphorylated TrkB at specific tyrosines to total TrkB, and are shown graphically as the percent of the sham VNS or vehicle value in the VNS studies and as the percentage of the control saline value for the antidepressant studies. Total TrkB did not change after either acute or chronic VNS or drugs as shown by the ratio of total TrkB/β-actin. Similarly, the ratios of phosphorylated Akt and ERK at specific amino acid residues to total Akt and ERK, respectively, are shown graphically as the percentage of the respective control group as mentioned above.

### Statistical analysis

Immunoblot bands were quantified using NIH ImageJ1.32. In experiments without K252a, data were analyzed using one-way multivariate analysis of variance (MANOVA) followed by Student's Newman-Keuls *post-hoc* tests. In experiments with K252a pretreatment, data were analyzed using a two-way MANOVA followed by Student's Newman-Keuls *post-hoc* tests. MANOVA was used as all three phosphorylation sites reside on the same protein and therefore are not independent measures. VNS data were analyzed separately from drug data. P <0.05 was considered significant. All data are presented as the mean ^+^/− SEM percentage of control values.

## Results

**Figure 1:**
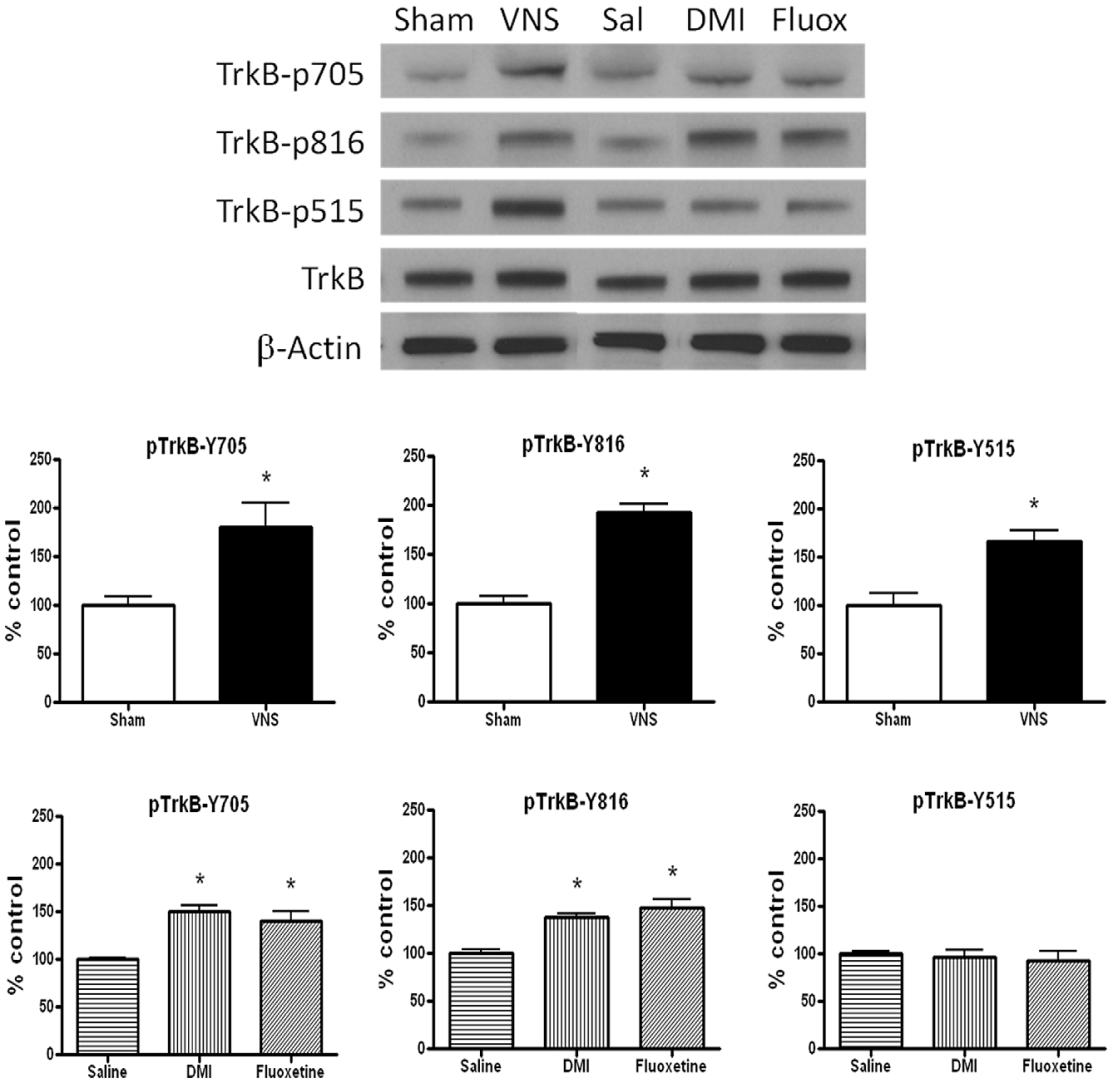
Vagal nerve stimulation (VNS) rapidly induces TrkB activation in rat hippocampus. Effects of acute (2 hr) VNS, desipramine (DMI, 10 mg/kg, i.p) or fluoxetine (15 mg/kg, i.p) on TrkB phosphorylation at different tyrosine residues in rat hippocampus.Phospho-TrkB values are normalized against total-TrkB values. One-way MANOVA, Student's Newman-Keuls *post-hoc* test, *P<0.05, n = 4−8 per group.

In the first experiment, the effect of acute (2 hr) VNS on TrkB phosphorylation was compared with that produced by acute administration of a selective serotonin reuptake inhibitor (SSRI), fluoxetine (15 mg/kg, i.p), and a selective norepinephrine reuptake inhibitor (NRI), desipramine (10 mg/kg, i.p) (Figure 1). The drugs were administered 2 hours prior to obtaining brain tissue. Multivariate ANOVAs were carried out separately either for VNS treatment or for drug treatment. For the antidepressant data, there was a significant main effect of treatment for tyrosine 705 [F(2,9) = 17.35, p<0.001] and for tyrosine 816 [F(2,9) = 17.35, p<0.001] but not for tyrosine 515 [F(2,9) = 0.28, p>0.05]. As expected [13], [14], *post-hoc* analysis revealed that acute administration of either fluoxetine or DMI caused a significant increase in phosphorylation of tyrosines 705 and 816 in the hippocampus compared to that measured in vehicle-treated rats (Figure 1). Acute administration of VNS did this also. Unexpectedly, acute VNS also significantly increased the phosphorylation of tyrosine 515 [F(1,13) = 16.21, p<0.01] (Figure 1). Total TrkB protein levels were not altered after either acute antidepressant or VNS treatment (Figure 1), e.g., the ratio of total TrkB/β-actin for the sham group was not significantly different from that in the VNS group (100^+^/−1.25 vs. 104.03^+^/−4.87, respectively, P>0.05).

**Figure 2:**
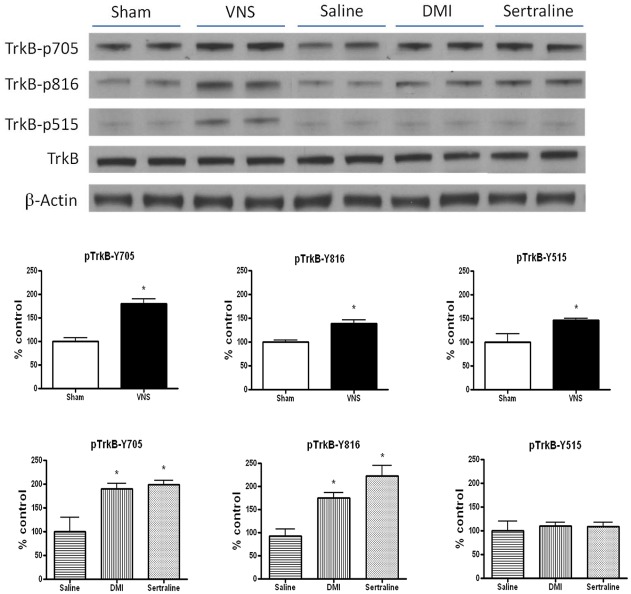
Vagal nerve stimulation (VNS) induces TrkB activation in rat hippocampus. Effects of chronic (14 days) VNS, desipramine (DMI, 10 mg/kg/day, i.p) or sertraline (7.5 mg/kg/day, i.p) on TrkB phosphorylation at different tyrosine residues in rat hippocampus.Phospho-TrkB values are normalized against total-TrkB values. One-way MANOVA, Student's Newman-Keuls *post-hoc* test, *P<0.05, n = 6−10 per group.

Given the importance of prolonged antidepressant and VNS treatments for optimal clinical response in depression, the next experiment examined the effect of longer administration, for 14 days, of VNS, sertraline (7.5 mg/kg/day, i.p) or DMI (10 mg/kg/day, i.p) on the phosphorylation of TrkB. The results were similar to what was seen acutely, namely the drugs and VNS significantly increased phosphorylation at Y705 [for drugs, F(2,15) = 7.98, p<0.01; for VNS, F(1,16) = 38.97, p<0.001] and Y816 [for drugs, F(2,15) = 15.39, p<0.001]; for VNS, F(1,16) = 10.58, p<0.01] but only VNS increased phosphorylation of Y515 [F(1,16) = 8.83, p<0.01] (Figure 2). Western blot analyses revealed that total TrkB protein levels were not altered after either chronic antidepressant or VNS treatment when compared with that measured in the control group (Figure 2).

**Figure 3:**
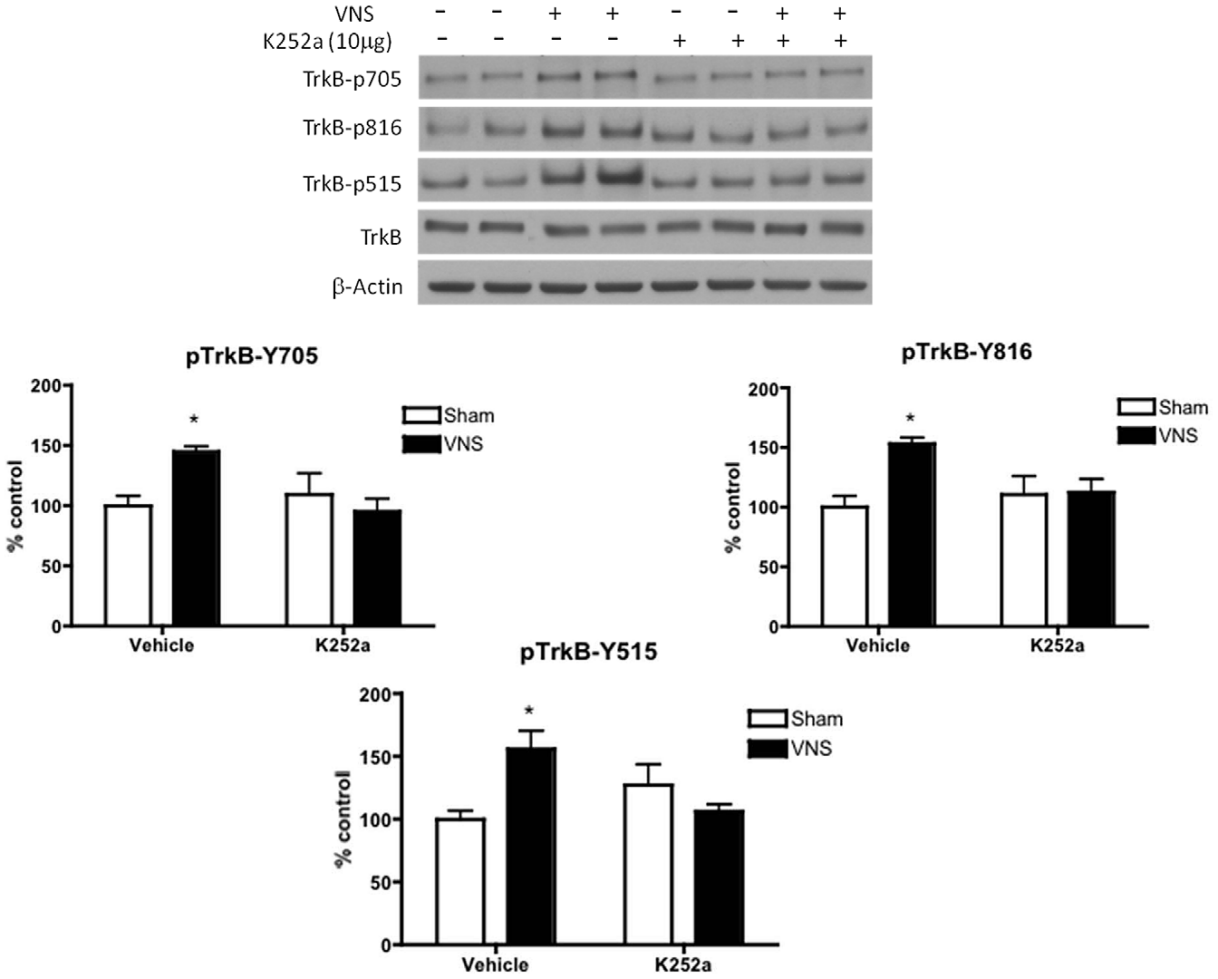
K252a inhibits VNS-induced TrkB phosphorylation in rat hippocampus. The ability of acute (2 hr) VNS to induce phosphorylation of TrkB was abolished by prior i.c.v administration ofK252a. Phospho-TrkB values are normalized against total-TrkB values. Two-way MANOVA, Student's Newman-Keuls *post-hoc* test, *P<0.05, n = 4−6 per group.

Since VNS is able to induce phosphorylation of TrkB at all three tyrosines (i.e. tyrosines 705, 816 and 515) and that the phosphorylation of tyrosine 515 is distinct from the effect of antidepressant drugs, there may be some differences in the mechanisms by which VNS and antidepressant drugs cause such effects. To determine whether VNS was causing tyrosine 515 to be phosphorylated through a mechanism not directly involving TrkB activation, i.e., not involving the autophosphorylation site, we tested the effect of an agent that prevents tyrosine kinase activity, K252a. It was administered intracerebroventricularly for 2 hours before the start of VNS, which was then given for 2 hours. K252a pretreatment blocked the effects of VNS on TrkB phosphorylation at all three tyrosines, namely Y705, Y816 and Y515 (Figure 3). Two-factor MANOVA showed a significant interaction between inhibitor x treatment for tyrosine 705 [F(1,14) = 6.98, p<0.05] for tyrosine 816 [F(1,14) = 5.24, p<0.05], and for tyrosine 515 [F(1,14) = 12.61, p<0.01]. *Post-hoc* analysis revealed that administration of K252a alone did not alter TrkB phosphorylation in comparison to that measured in rats pretreated with vehicle. Total TrkB protein levels were not altered (Figure 3).

**Figure 4:**
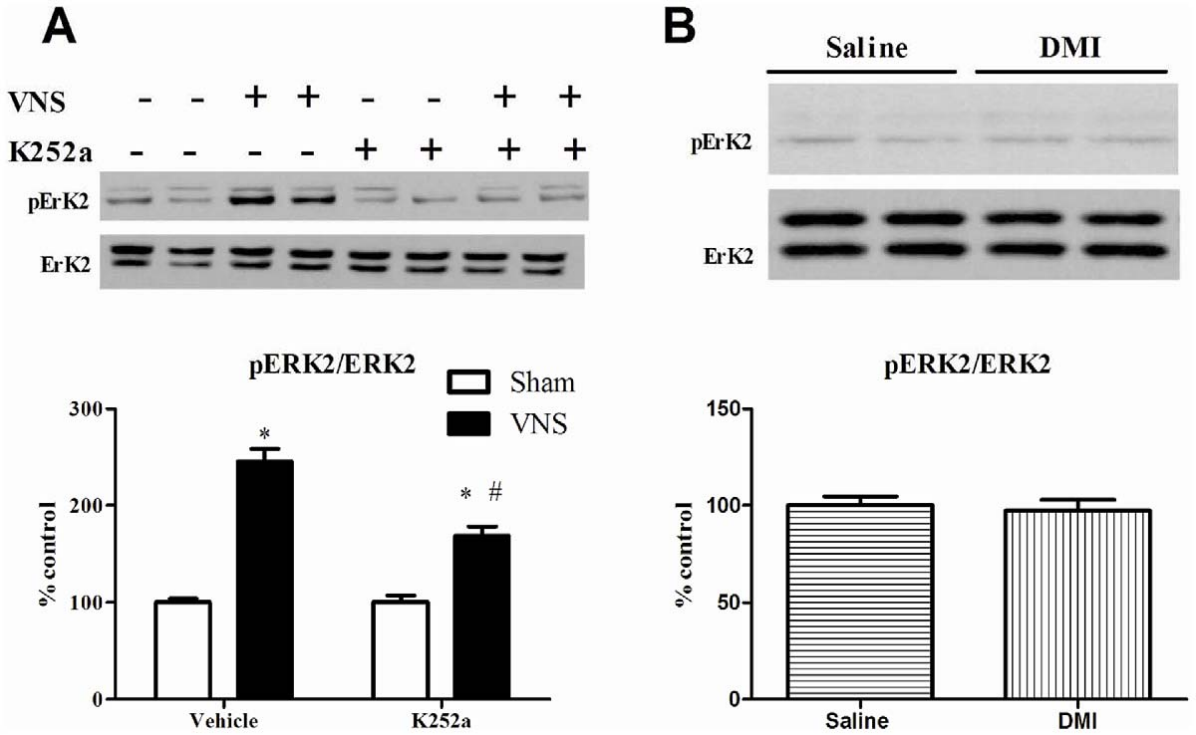
Effects of acute (2 h) VNS (A) or DMI administration (B) on ERK phosphorylation in rat hippocampus. Phospho-ERK2 values are normalized against total ERK2 values. Data were analyzed by two-way ANOVA followed by Student's Newman-Keuls *post-hoc test*. *Significantly different from corresponding control value, P<0.05. #Significantly different from VNS value in vehicle group, P<0.05, n = 7 per group for VNS and 6 per group for DMI. The upper bands seen in the representative images correspond to pERK1 or ERK1 and were not analyzed due to poor immunoreactivity of pERK1.

Because of the VNS-induced phosphorylation of Y515, subsequent experiments compared the effect of VNS to that produced by DMI on phosphorylation of proteins downstream of the 515 site on TrkB, namely Akt and ERK2 [2], [32]. As shown in Figure 4A, VNS given for two hours significantly increased the phosphorylation of pERK2 and this effect was reduced, but not completely blocked, by prior i.c.v administration of K252a. The results of the two-way ANOVA analysis showed a significant main effect of VNS [F(1, 24) = 145.5, p<0.001] and K252a [F(1, 24) = 18.5, p<0.001] as well as a significant interaction between VNS and K252a [F(1, 24) = 19.14, p<0.001]. By contrast, DMI (10 mg/kg, i.p) treatment did not significantly increase pERK2 (p = 0.72) (Fig. 4B).

**Figure 5:**
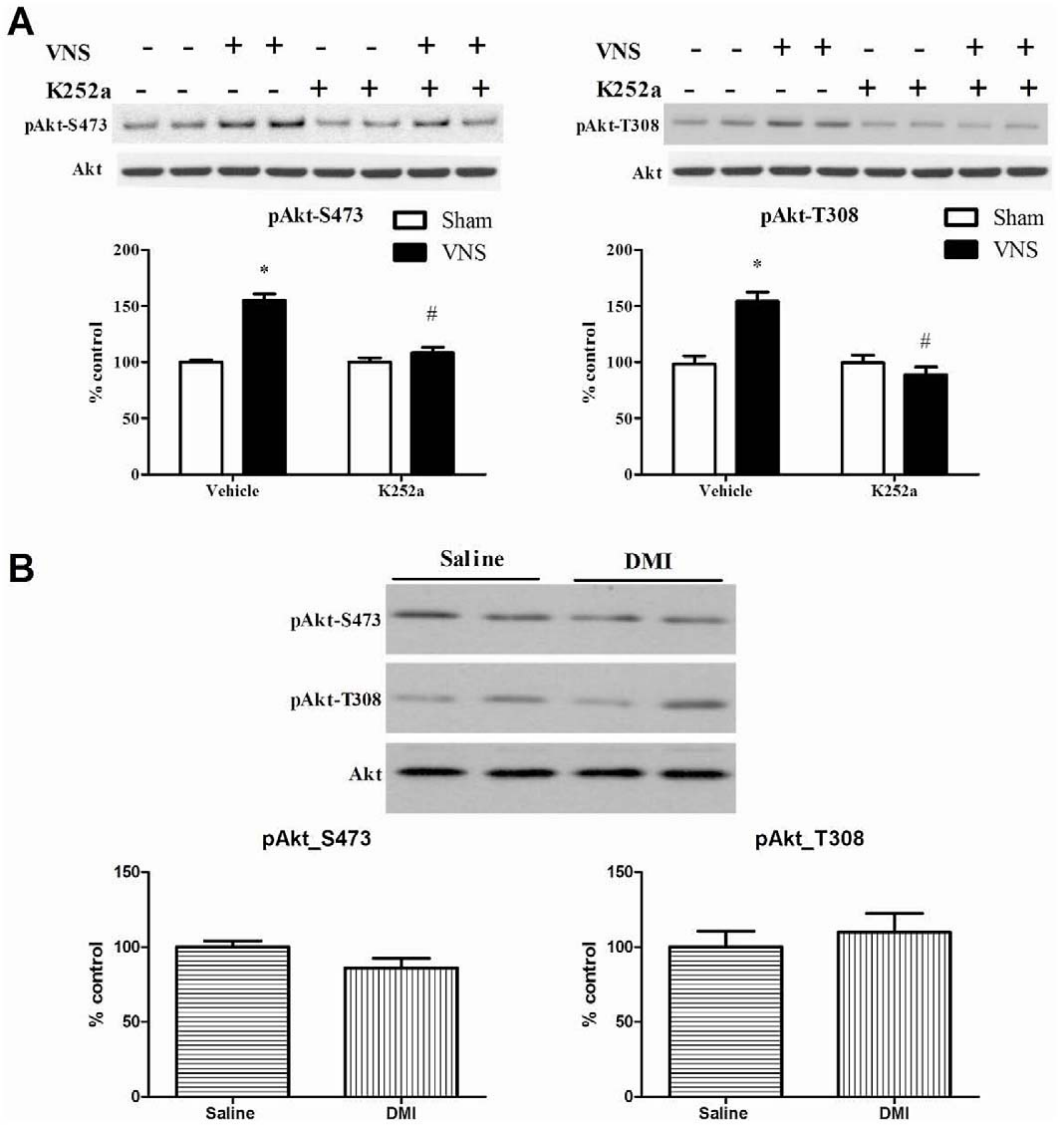
Effects of acute (2 h) VNS (A) or DMI administration (B) on Akt phosphorylation (S473 and T308) in rat hippocampus. Phospho-Akt values are normalized against total Akt values. Data were analyzed by two-factor MANOVA followed by Student's Newman-Keuls *post-hoc test*. *Significantly different from corresponding control value, P<0.05. #Significantly different from VNS value in vehicle group, P<0.001; n = 7 per group for VNS and 6 per group for DMI. No significant main effect was obtained for DMI.

Similar results were obtained for the two phosphorylation site sites on Akt, namely S475 and T308 (Figure 5A). Two-factor MANOVA analysis revealed a significant main effect of VNS [for S475, F(1, 24) = 49.38, p<0.001; for T308, F(1, 24) = 17.50, p<0.001] as well as K252a [for S475, F(1, 24) = 26.82, p<0.001; for T308, F(1, 24) = 34.38, p<0.001]. A significant interaction was also found between VNS and K252a [for S475, F(1, 24) = 26.82, p<0.001; for T308, F(1, 24) = 34.38, p<0.001]. *Post hoc* analysis indicated VNS caused significant increases in phosphorylation of both S475 and T308. In this case, though, K252a produced a complete, rather than partial, blockade of the effect of VNS (Figure 5A). As before, DMI did not significantly increase phosphorylation of either residue [for S475, F(1, 10) = 3.31, p>0.05; for T308, F(1, 10) = 0.35, p = 0.56] (Figure 5B).

## Discussion

The most interesting finding in this study is that both acute and chronic VNS administration not only increased phosphorylation of the TrkB receptor at Y705 and Y816 but at Y515 also. In confirmation of previous work carried out using mice [13], [14], we found also in rats that different types of antidepressants increased phosphorylation of this receptor at Y707 and Y816 but not at Y515. Thus, among antidepressant treatments evaluated to date, the phosphorylation of Y515 caused by VNS is unique. Similar TrkB protein levels were observed across all treatment groups when compared with control groups thus discounting the possibility that changes in phosphorylation were due to alterations in TrkB protein levels. Stimulation of the TrkB receptor activates several intracellular signaling cascades that can be regulated independently of each other; among them, the MAPK, PI3K and PLC-γ1 pathways are best characterized [2]. The present study revealed that acute and chronic treatments of rats with VNS increased TrkB receptor phosphorylation at sites that can activate PLC-γ1 and MAPK/PI3K signal transduction pathways. Pretreatment with K252a blocked this effect of VNS. Consistent with the phosphorylation of Y515, VNS increased phosphorylation of its downstream effectors ERK and Akt and this effect was also reduced or blocked by K252a. By contrast, both acute and chronic administration of standard antidepressants such as sertraline and DMI induced phosphorylation of TrkB receptor at the site linked to PLC-γ1 signaling without altering the MAPK/PI3K pathway and DMI treatment did not increase phosphorylation of ERK or Akt.

Both acute and chronic VNS have been found to increase mRNA levels of BDNF [23] and BDNF protein [24] in rat hippocampus. However, as the majority of brain BDNF is intracellular [33], total brain BDNF does not reliably reflect its release. Only a small amount of total tissue BDNF is released upon activation of neurons [33], [34] such that only a small fraction is available to activate TrkB receptors. Consequently, to provide another measure of drug effects on BDNF function, the phosphorylation state of the TrkB receptor has been measured using Western blot analysis with site-directed antibodies. Changes in the phosphorylation status of TrkB occur upon antidepressant administration to mice. Saarelainen et al. [14] and Rantamaki et al. [13] reported that 30–120 min after administration of either imipramine, fluoxetine, citalopram, reboxetine or moclobemide, phosphorylation of TrkB at Y705 and Y816 in mouse brain increased significantly. The initial report [14] had this effect being most pronounced in the anterior cingulate cortex rather than in the hippocampus but the subsequent report [13] found it in the hippocampus as well. Similar to what was observed in mice, the present study indicates that acute administration of fluoxetine or DMI also increased phosphorylation of TrkB at these tyrosines in rat hippocampus. In addition, acute VNS administration also significantly increased phosphorylation of TrkB at Y515. Thus, both acute administration of standard antidepressants and VNS phosphorylate TrkB at a site linked to increased PLC-γ1 signaling, whereas only acute VNS phosphorylated the tyrosine linked to MAPK/PI3K signal transduction.

Further experiments were carried out to examine downstream signaling linked to Y515 such as phosphorylation of both Akt (at serine 473 and threonine 308) and ERK1/2 (at threonine 202 for ERK1 and Tyr204 for ERK2) in total hippocampus. VNS treatment consistently caused an increase in phosphorylation of both Akt and ERK2, whereas treatment with DMI failed to increase in Akt and ERK phosphorylation. Further studies using immunohistochemical detection instead of western blot analysis is needed to address whether or not DMI could potentially cause a more selective pattern of activation of specific subregions within hippocampus.

If BDNF were mediating the effect of antidepressants, one might expect all three tyrosines to be phosphorylated. It is often assumed that the signaling molecule causing the increase in antidepressant-induced phosphorylation of TrkB is BDNF. However, a recent study by Rantamaki et al. [35] indicates that antidepressants activate TrkB receptors in the mouse brain in a manner independent of BDNF. In particular, they showed that in conditional mutant mice lacking BDNF in the forebrain, acute administration of imipramine was still able to cause phosphorylation of Y816, albeit to a lesser extent than that in wild-type mice. It was concluded that antidepressants transactivate TrkB receptors in forebrain independently of BDNF. This conclusion is perhaps not surprising since, in cells in culture, BDNF causes phosphorylation of TrkB at all three sites [36], [37] and stimulates signal transduction pathways mediated by activation of Y816 and Y515 [36], [37], [38]. Also, infusion of BDNF into the dentate gyrus of rats increased activation of ERK2 at 15 min and 3 hours [39].

However, as VNS increases phosphorylation of all three tyrosines and, in addition, induces the phosphorylation of Akt/ERK known to be coupled to Y515, it still remains possible that BDNF (and/or NT-4) is mediating its effect on TrkB. In addition, it is possible that VNS increases TrkB signaling by mechanisms in addition to its influencing BDNF or NT-4 release. Increased intracellular cAMP [40], [41] or membrane depolarization [41], [42] have been shown to increase membrane surface expression of TrkB thus enhancing the ability of BDNF to induce TrkB autophosphorylation. Furthermore, some G-protein-coupled receptors have been shown to transactivate TrkB receptors independently of BDNF [43], [44], [45]. Finally, as NT-3 (and NT-4/5) is a low-affinity ligand for TrkB, VNS may induce TrkB activation by enhancing the effects of this neurotrophin [46]. Whether these molecular mechanisms are involved in VNS-induced TrkB activation remains to be elucidated.

It is also possible that the effects of VNS could be ligand-mediated, whereas the effects of DMI could potentially be mediated by transactivation of TrkB through G-protein coupled receptors (GPCRs). DMI is a tricyclic antidepressant that preferentially blocks norepinephrine transporters [47] leading to enhanced noradrenergic activity and activation of noradrenergic receptors. It is known that GPCRs can directly activate ERK/MAP kinases [48] or mediate transactivation of Trks in the central nervous system [44], [49]. With regard to the hippocampus, studies *in vitro* have shown that norepinephrine application in cultured hippocampal neurons is able to induce TrkB phosphorylation and downstream signaling (activation of ERK and PI3-K) via GPCR transactivation of TrkB [50]. However, that study has not addressed specifically which tyrosine sites were activated within TrkB and also differ from our *in vivo* approach. Cultured neurons lack all the physiological inputs that could be responsible for the mechanisms by which TrkB is differentially activated.

Other possible mechanisms underlying this difference between the effects of the antidepressants and VNS include membrane surface expression of TrkB isoforms. Alternative splicing of the TrkB pre-mRNA from its locus on DNA yields two isoforms [51]. One is a full-length form of TrkB, TK+, which has the cytosolic tyrosine kinase domain. The other is the tyrosine kinase lacking isoform, TK−, which consists of two isoforms, T1 and T2. These truncated isoforms contain the same extracellular and transmembrane domains but different C-terminal sequences. T1 is capable of binding to BDNF to the same level as TK+ [52] and is hypothesized to block TK+ functions, such as TK+ phosphorylation, by forming heterodimers with TK+ [53], [54]. Transgenic mice over-expressing T1 receptors in brain have reduced TrkB phosphorylation and are resistant to effects of antidepressants in the FST [14]. These data indicate that activation of TrkB plays a role in the antidepressant-induced behavioral effects and that T1 is able to block such functions. Moreover, T1 has been shown to have its own signaling pathway that involves the PLC-IP3 pathway [55]. In addition, there is a difference in intracellular localization of TK+ and T1. In adult brains, TK+ is localized in both pre- and postsynaptic regions [56], [57], [58] whereas T1 is concentrated in the presynaptic region [56], [57]. Du et al. [59] showed that electrical stimulation facilitated the movement of TrkB from the intracellular pool to the cell surface, particularly in dendrites. Trk receptors in signaling endosomes activate signaling events that are different from those activated on the cell surface [60], [61]. Hence, it is possible that antidepressants and VNS produce different activity-dependent enhancement of TrkB signaling.

K252a, an alkaloid-like compound isolated from *Nocardiopsis*, is frequently used as a nonspecific protein kinase inhibitor for blocking Trk activity. It was first described as an inhibitor of protein kinase C [62]. However, Koizumi et al. [63] demonstrated that it could inhibit Trk-dependent actions at a concentration that did not block the PKC-dependent action. A subsequent paper established that K252a could inhibit tyrosine kinase activity of Trk receptors [64]. K252a also inhibits other classes of tyrosine kinase receptors, as well as Ser/Thr kinases, including those in the MAPK cascade [65]. K252a and its analogs appears to interact directly with the tyrosine-specific protein kinase domain of the neurotrophin receptors [29]. K252a has been shown to block autophosphorylation of TrkB and inhibit many biological functions of BDNF [30]. When administered intracerebroventricularly at the same dose we used, Benmansour et al. [31] found that it blocked the inhibitory effect of exogenously administered BDNF on serotonin clearance, as measured by chronoamperometry. Interestingly, Shirayama et al. [66] showed that local administration of K252a into the hippocampus blocked the antidepressant-like effects of BDNF, also locally administered into the hippocampus, in the learned helplessness paradigm. Similarly, we found that i.c.v administration of K252a blocked the effects of VNS on TrkB phosphorylation. Whether inhibition of TrkB phosphorylation by K252a would interfere with behavioral effects of VNS is an important outstanding question.

In conclusion, VNS phosphorylates tyrosine 515 on TrkB and also the activation of effectors coupled to this Y515 site (Akt/ERK), and this effect is distinct from those phosphorylated by standard antidepressant drugs. What role, if any, this plays in the behavioral effect of VNS remains to be clarified by future experiments.
